# Regulation of Dopamine Uptake by Vasoactive Peptides in the Kidney

**DOI:** 10.1155/2016/6302376

**Published:** 2016-08-22

**Authors:** N. L. Rukavina Mikusic, N. M. Kouyoumdzian, E. Rouvier, M. M. Gironacci, J. E. Toblli, B. E. Fernández, M. R. Choi

**Affiliations:** ^1^Instituto de Investigaciones Cardiológicas ININCA, UBA-CONICET, Facultad de Farmacia y Bioquímica, UBA, Buenos Aires, Argentina; ^2^Cátedras de Anatomía e Histología, Facultad de Farmacia y Bioquímica, UBA, Buenos Aires, Argentina; ^3^Cátedras de Química Biológica, Facultad de Farmacia y Bioquímica, UBA, Buenos Aires, Argentina; ^4^Laboratorio de Medicina Experimental, Hospital Alemán, Buenos Aires, Argentina

## Abstract

Considering the key role of renal dopamine in tubular sodium handling, we hypothesized that c-type natriuretic peptide (CNP) and Ang-(1-7) may regulate renal dopamine availability in tubular cells, contributing to Na^+^, K^+^-ATPase inhibition. Present results show that CNP did not affect either ^3^H-dopamine uptake in renal tissue or Na^+^, K^+^-ATPase activity; meanwhile, Ang-(1-7) was able to increase ^3^H-dopamine uptake and decreased Na^+^, K^+^-ATPase activity in renal cortex. Ang-(1-7) and dopamine together decreased further Na^+^, K^+^-ATPase activity showing an additive effect on the sodium pump. In addition, hydrocortisone reversed Ang-(1-7)-dopamine overinhibition on the enzyme, suggesting that this inhibition is closely related to Ang-(1-7) stimulation on renal dopamine uptake. Both anantin and cANP (4-23-amide) did not modify CNP effects on ^3^H-dopamine uptake by tubular cells. The Mas receptor antagonist, A-779, blocked the increase elicited by Ang-(1-7) on ^3^H-dopamine uptake. The stimulatory uptake induced by Ang-(1-7) was even more pronounced in the presence of losartan, suggesting an inhibitory effect of Ang-(1-7) on AT1 receptors on ^3^H-dopamine uptake. By increasing dopamine bioavailability in tubular cells, Ang-(1-7) enhances Na^+^, K^+^-ATPase activity inhibition, contributing to its natriuretic and diuretic effects.

## 1. Introduction

Renal dopamine plays a key role in sodium handling in order to achieve a normal salt and water balance [1]. Dopamine is synthesized in the kidney by proximal tubular cells and released in the tubular lumen to act as a paracrine hormone to promote natriuresis by inhibiting several sodium transporters such as apical Na^+^/H^+^ exchanger 3, Na^+^/Pi cotransporter, Na^+^/HCO_3_^−^ cotransporter, and basolateral Na^+^-K^+^-ATPase [2, 3].

Diverse vasoactive peptides regulate blood pressure levels through modulation of renal function. In this way, members of the natriuretic peptide system, such as atrial natriuretic peptide (ANP), brain natriuretic peptide (BNP), c-type natriuretic peptide (CNP), and urodilatin (URO), regulate sodium excretion in the kidney for maintenance of the extracellular volume [4]. CNP that was first isolated in porcine brain is a 22-amino acid peptide, with high homology with ANP and BNP, and lacks the carboxyterminal extension present in both peptides [5]. Although CNP is mainly produced by the vascular endothelium, it is also expressed in several tissues and cells, including the kidney [6, 7]. CNP exerts its actions through the specific binding to natriuretic peptide receptor type B (NPR-B), increasing intracellular cGMP levels in target cells [8]. In the last years, several studies have demonstrated the renal action of CNP [4]. The NPR-B receptor is expressed in different segments along the nephron [9]. Igaki et al. and Pham et al. reported that the infusion of CNP in rats increased the natriuresis and the fractional excretion of sodium [10, 11]. The urinary excretion of CNP is increased in patients with heart failure, suggesting a possible regulation of renal CNP synthesis by the systemic volume status [4].

Ang-(1-7) is a biologically active heptapeptide that represents the main component of the depressor and protective arm of the renin angiotensin system [12]. Besides of its well known actions as a protecting agent on cardiovascular system, Ang-(1-7) exhibits opposing actions to angiotensin II (Ang II), promoting natriuresis and diuresis [12, 13]. Ang-(1-7) effects in the kidney are mainly mediated by Mas receptors, although some actions may occur via AT1 or AT2 receptors [14]. Mas mRNA and Mas receptor have been detected mainly in proximal tubular cells by different techniques [13]. Experimental evidences demonstrate that the administration of Ang-(1-7) increased the urinary flow rate and sodium excretion [15, 16]. These natriuretic and diuretic actions of the peptide may be mediated by regulating the activity of sodium transporters in the proximal tubule, including the Na^+^-K^+^-ATPase [15].

Renal dopamine may come from neuronal and extraneuronal sources. The extraneuronal source includes the uptake of dopamine from the blood and the tubular fluid, where the organic cation transporters (OCTs and OCTNs) play an important role [17]. We have previously demonstrated that ANP and URO, through stimulation of dopamine uptake, favor dopamine intracellular accumulation which in turn results in an overinhibition of Na^+^, K^+^-ATPase activity [18, 19]. This process is mediated by the NPR-A-cGMP-PKG intracellular pathway [20, 21]. As a physiological antagonist, Ang II exhibits the opposite effect through AT1R and PKA and cAMP as second messenger [22, 23].

Considering that CNP and Ang-(1-7) exert natriuretic and diuretic actions, we hypothesize that these peptides might regulate dopamine uptake by the tubular cells, controlling its bioavailability to bind D1 receptors. Therefore, both vasoactive peptides might collaborate with dopamine to enhance the inhibition of Na^+^, K^+^-ATPase activity and to promote a greater natriuresis. In this sense, the aim of the present study was to investigate whether CNP and Ang-(1-7) could regulate dopamine uptake and Na^+,^ K^+^-ATPase activity in renal tissue samples.

## 2. Materials and Methods

### 2.1. Animals

Male Sprague-Dawley rats (10–12 weeks old, 250–350 g of body weight) were housed at controlled temperature (23 ± 2°C) and exposed to a daily 12-hour light-dark cycle (lights on from 07:00 a.m. to 07:00 p.m.) with free access to tap water and standard rat chow (Cooperación SRL, Argentina) until the day of the experiment. Experiments were conducted in accordance with the international guiding principles and local regulations regarding the care and use of laboratory animals for biomedical research as well as the “International Ethical Guiding Principles for Biomedical Research on Animals” established by the CIOMS (Council for International Organizations of Medical Sciences). The protocol was approved by the Institutional Committee for Care and Use of Laboratory Animals of the School of Pharmacy and Biochemistry of University of Buenos Aires (number: 2100-15, 0035638/15). All surgery was performed under ethyl urethane anaesthesia and all efforts were made to minimize suffering.

### 2.2. Drugs and Solutions

The following drugs and solutions were used in the experiments: ^3^H-dopamine and 28.0 Ci/mmol of specific activity (New England Nuclear, Boston, MA, USA); CNP, hydrocortisone, nomifensine, anantin, des (Gln 18-Ser 19-Gly 20-Leu 21-Gly 22) atrial natriuretic peptide fragment 4-23-amide, Ang-(1-7), losartan, A-779, PD123319, carbidopa, imidazole, ATP (adenosine 5′ triphosphate), bovine seroalbumin fraction V of Cohn, and Folin reactive were from Sigma-Aldrich, Inc., Saint Louis, Missouri, USA. EcoLite, for liquid scintillation, was from ICN Pharmaceutical Inc., CA, USA.

Standard Krebs bicarbonate (SKB) solution of the following composition (mM) was used as incubation medium: 118 NaCl; 4.7 KCl; 1.2 MgSO_4_·7H_2_O; 1.0 NaH_2_PO_4_; 2.4 CaCl_2_; 0.004 EDTA; 11.1 glucose; 0.11 ascorbic acid; 26.0 NaHCO_3_.

### 2.3. Procedures

Rats were anesthetized with 10% w/v ethyl urethane (1.2 mg/kg body weight, i.p.). Both kidneys were removed and washed with fresh SKB to remove the residual blood, and then slices of renal cortex and medulla were cut, minced, and weighed (approximately 40 mg).

### 2.4. Determination of ^3^H-Dopamine Uptake

^3^H-dopamine uptake was measured as previously described by us [20]. Briefly, tissues were placed in 2.0 mL SKB incubation medium in a Dubnoff incubator and preincubated at 37°C, pH 7.40, and bubbled with a gaseous mixture of 95% O_2_ and 5% CO_2_ for 15 min; nomifensine (50 *μ*M) was added in the preincubation medium to avoid neuronal dopamine uptake. Thereafter, tissues were transferred to fresh SKB and incubated, in similar conditions, with 0.625 *μ*Ci/mL of ^3^H-dopamine (22.32 nM), 50 *μ*M nomifensine, and the different inhibitors for 15 min. After that time, CNP or Ang-(1-7) was added to the medium and the incubation continued for another 30 min. Control groups were incubated in the absence of the peptides. The following experiments were carried out in samples of renal cortex.

### 2.5. CNP and Ang-(1-7) Effects on ^3^H-Dopamine Uptake

A concentration-response curve to CNP and Ang-(1-7) (1 pM to 100 nM) was performed to examine their effects on ^3^H-dopamine uptake. The following groups were studied: control and incubated with 1, 10, and 100 pM and 1, 10, and 100 nM of CNP or Ang-(1-7).

A time-course curve was carried out to study CNP and Ang-(1-7) effects on ^3^H-dopamine uptake at different times (5, 10, 15, 20, and 30 minutes). The following groups were studied: control and incubated with 100 nM of CNP or 100 nM of Ang-(1-7).

To investigate CNP and Ang-(1-7) effects on ^3^H-dopamine uptake in different areas of the kidney, ^3^H-dopamine uptake was determined in samples from renal cortex and medulla in the following groups: control and incubated with 100 nM of CNP or 100 nM of Ang-(1-7).

### 2.6. Identification of CNP and Ang-(1-7) Receptors

The following groups were studied in order to analyze if NPR-A or NPR-C receptors were involved in CNP effects on ^3^H-dopamine uptake: (a) control and incubated with (b) 100 nM CNP; (c) 100 nM anantin (specific NPR-A receptor blocker); (d) 100 nM anantin plus 100 nM CNP; (e) 100 nM cANP (4-23-amide) (specific NPR-C receptor agonist); (f) 100 nM cANP (4-23-amide) plus 100 nM CNP.

To analyze if AT1R, AT2R, or Mas receptor was involved in Ang-(1-7) effects on ^3^H-dopamine uptake, the following groups were studied: (a) control and incubated with (b) 100 nM Ang-(1-7); (c) 100 nM A-779 (specific Mas receptor blocker); (d) 100 nM A-779 plus 100 nM Ang-(1-7); (e) 100 nM PD123319 (specific AT2R blocker); (f) 100 nM PD123319 plus 100 nM Ang-(1-7); (g) 100 nM losartan (specific AT1R blocker); (h) 100 nM losartan plus 100 nM Ang-(1-7).

At the end of the incubation period, the tissues were washed with cold SKB solution, along 3 periods of 5 min each, and then homogenized with 2.5 mL of 10% trichloroacetic acid. The homogenates were centrifuged at 5000 rpm (1700 g) at 4°C for 30 min and tritium activity in the supernatants was determined by usual scintillation counting method. Results of ^3^H-dopamine uptake are expressed as d.p.m./g of fresh tissue.

### 2.7. Effects of CNP and Ang-(1-7) on Na^+^, K^+^-ATPase Activity

To test whether the increase in renal dopamine produced by CNP or Ang-(1-7) could be associated with changes in Na^+^, K^+^-ATPase activity, the following experiments were performed in the presence of nomifensine (to avoid neuronal dopamine uptake) and carbidopa (to avoid dopamine synthesis). Carbidopa was administered* in vivo* (200 *μ*g/kg, i.p., 24 and 2 h before sacrifice) and* in vitro* (100 M in the medium, along the preincubation and incubation periods). CNP as well as Ang-(1-7) effects were tested in the presence and in the absence of radio unlabeled dopamine and the nonneuronal dopamine uptake blocker hydrocortisone.

The following groups were studied: (a) control and (b) incubated with 100 *μ*M carbidopa. The next groups were under 100 *μ*M carbidopa: (c) 1 *μ*M dopamine; (d) 100 nM CNP; (e) 100 nM Ang-(1-7); (f) 100 *μ*M hydrocortisone; (g) 100 *μ*M hydrocortisone plus 1 *μ*M dopamine; (h) 100 nM CNP plus 1 *μ*M dopamine; (i) 100 nM Ang-(1-7) plus 1 *μ*M dopamine; (j) 100 nM CNP plus 1 *μ*M dopamine plus 100 *μ*M hydrocortisone; (k) 100 nM CNP plus 1 *μ*M dopamine plus 100 *μ*M hydrocortisone.

Tissues were incubated for 30 min as described above and then homogenized (1 : 10 weight/volume) in 25 mM imidazole-1 mM EDTA-0.25 M sucrose solution and centrifuged at 5000 rpm (1700 g) at 4°C for 15 min. Na^+^, K^+^-ATPase activity was assayed in the supernatant as previously described [20]. ATPase activity was measured by colorimetric determination of released orthophosphate [24, 25]. Protein concentrations were determined by the method of Lowry et al. [26]. Results are expressed as percentage of Na^+^, K^+^-ATPase activity, considering control values as 100%.

### 2.8. Statistical Analysis

All values are expressed as mean ± SEM. Data were processed using GraphPad in Stat Software (San Diego, CA, USA). Student's* t*-test, one-way analysis of variance (ANOVA), and the Tukey test were performed when they corresponded. *p* values of 0.05 or less were considered statistically significant.

## 3. Results

### 3.1. Effects of CNP and Ang-(1-7) on ^3^H-Dopamine Uptake

**Figure 1:**
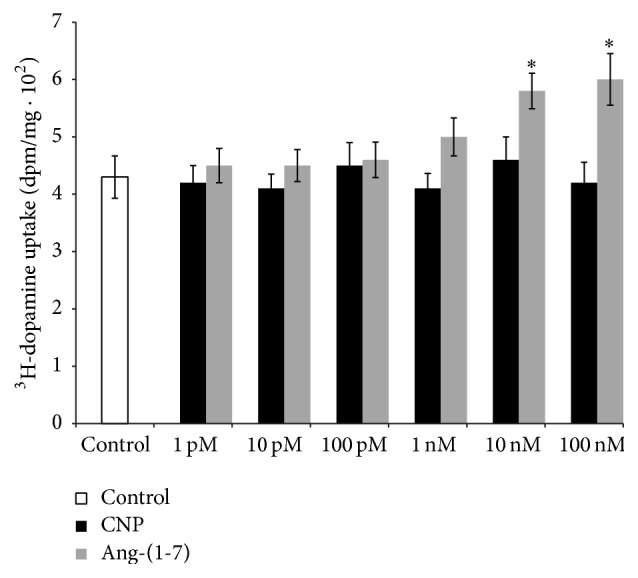
Effects of increasing concentrations (1 pM–100 nM) of CNP and Ang-(1-7) on ^3^H-dopamine uptake in experiments carried out* in vitro* in isolated renal cortex. ^3^H-dopamine uptake is expressed as dpm/mg ± SEM. ^*∗*^ *p* < 0.05 compared with control. Number of samples: 8–10.

Figure 1 shows the effects of increasing concentrations of CNP and Ang-(1-7) (1 pM to 100 nM) on ^3^H-dopamine uptake in renal cortex. 10 nM and 100 nM Ang-(1-7) caused a significant increase in ^3^H-dopamine uptake. Then, we used the up threshold concentration of 100 nM Ang-(1-7) to continue the studies. On the other hand, CNP at any concentration used failed to alter ^3^H-dopamine uptake.

**Figure 2:**
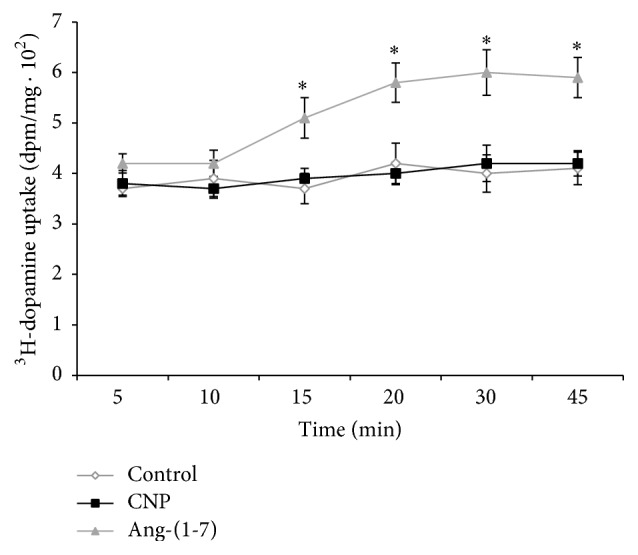
Effects of 100 nM CNP and 100 nM Ang-(1-7) on the time-course curve of ^3^H-dopamine uptake in isolated renal cortex samples, between 5 and 45 min. ^3^H-dopamine uptake is expressed as dpm/mg ± SEM. ^*∗*^ *p* < 0.01 compared with control. Number of samples: 8–10.

The time course of ^3^H-dopamine uptake between 5 and 45 min is displayed in Figure 2. 100 nM Ang-(1-7) increased dopamine uptake at 15 min and this effect lasted up to 45 min with a maximum difference at 30 minutes when compared with control group. Therefore, we carried out further studies on ^3^H-dopamine uptake with 30-minute incubation period. On the contrary, CNP did not affect ^3^H-dopamine uptake at any time.

**Figure 3:**
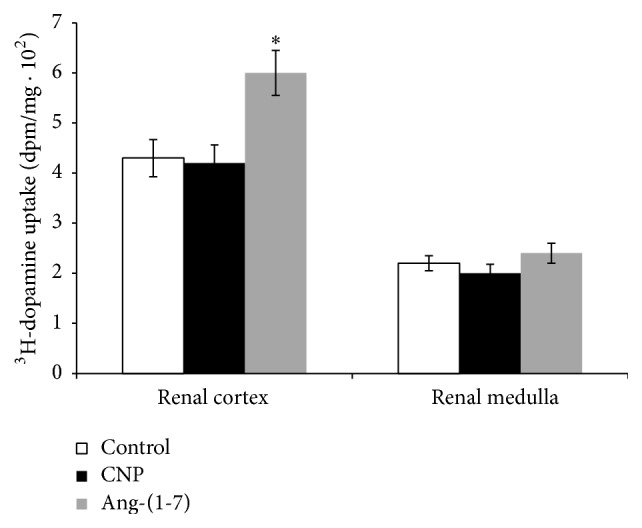
Effects of 100 nM CNP and 100 nM Ang-(1-7) on ^3^H-dopamine uptake in experiments carried out* in vitro* in isolated renal cortex and medulla samples. ^3^H-dopamine uptake is expressed as dpm/mg ± SEM. ^*∗*^ *p* < 0.05 compared with control. Number of samples: 8–12.

Figure 3 shows that 100 nM Ang-(1-7) increased ^3^H-dopamine uptake in renal cortex but lacked effects in renal medulla. CNP was not able to modify ^3^H-dopamine uptake of both renal cortex and medulla.

**Figure 4:**
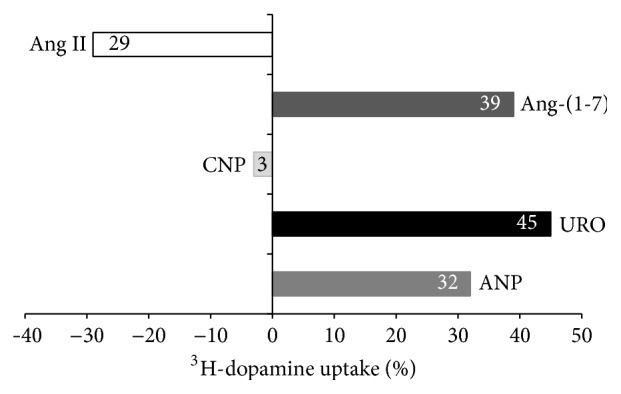
Comparison of different vasoactive peptides effects on ^3^H-dopamine uptake in experiments carried out* in vitro* in isolated renal cortex samples. ^3^H-dopamine uptake is expressed as percentage of uptake respect control group.

Considering that both ANP and URO increased ^3^H-dopamine uptake in renal cortex [18, 19], and Ang II exhibited the opposite effect [22], we proceeded to compare the potency of these vasoactive peptides (each used in equimolar concentrations of 100 nM and expressed as percentages of modification of dopamine uptake) on dopamine uptake with the effects obtained in the present work by CNP and Ang-(1-7).  Figure 4 illustrates that URO displayed the maximum increasing effect of 45% versus 39% of ANG 1-7 and 32% of ANP, while ANG II decreased dopamine uptake by 29%. The effect of CNP was not significant.

### 3.2. Identification of CNP and Ang-(1-7) Receptors

**Figure 5:**
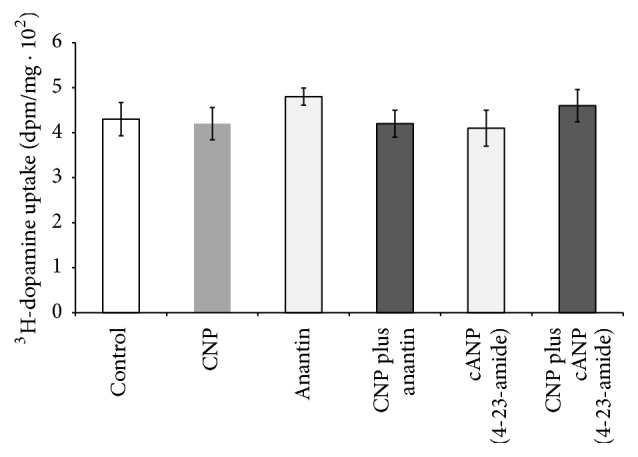
Effects of 100 nM CNP in the presence of 100 nM anantin or 100 nM cANP (4-23-amide) on ^3^H-dopamine uptake in renal cortex. ^3^H-dopamine uptake is expressed as dpm/mg ± SEM. Number of samples: 8–11.

The next goal was to test whether CNP effects on dopamine uptake were mediated by activation of NPR-B receptors. Since CNP acts mainly through activation of NPR-B receptors, we wanted to discard that the lack of effects of CNP on dopamine uptake, shown in Figure 1, could be masked by activation of NPR-A by CNP. Thus, we tested the action of CNP on ^3^H-dopamine uptake when NPR-A receptors were blocked by the selective NPR-A receptor blocker, 100 nM anantin. In Figure 5, it can be observed that CNP did not affect ^3^H-dopamine uptake, suggesting that neither activation of NPR-A nor activation of NPR-B by CNP can affect this process. Moreover, the specific NPR-C agonist, 100 nM cANP (4-23-amide), neither altered dopamine uptake nor modified the effect of CNP on dopamine uptake.

**Figure 6:**
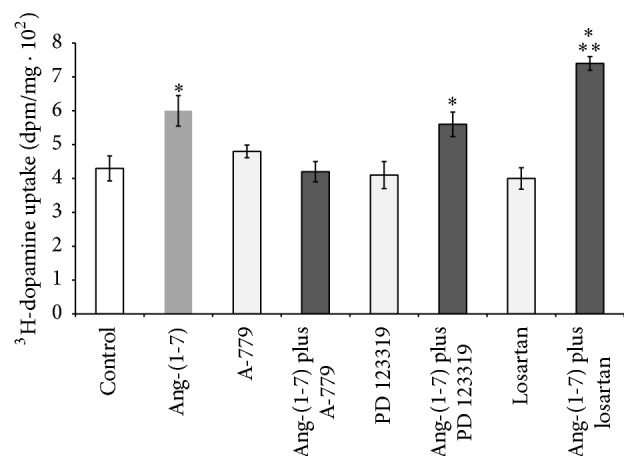
Effects of 100 nM Ang-(1-7) in the presence of 100 nM A-779 or 100 nM PD123319 or 100 nM losartan on ^3^H-dopamine uptake in renal cortex. ^3^H-dopamine uptake is expressed as dpm/mg ± SEM. ^*∗*^ *p* < 0.05 compared with control. ^*∗∗*^ *p* < 0.05 compared with Ang-(1-7) or Ang-(1-7) plus PD123319. Number of samples: 8–12.

In order to determine the receptor subtype involved in the stimulatory activity of Ang-(1-7) on ^3^H-dopamine uptake in renal cortex, we proceeded to evaluate the participation of AT1R, AT2R, and Mas receptor by using different specific antagonist (Figure 6). The selective Mas receptor blocker, 100 nM A-779, blocked 100 nM Ang-(1-7) effect on dopamine uptake, suggesting the involvement of Mas receptor. In contrast, the specific AT2R antagonist, 100 nM PD123319, did not modify the stimulatory effect of Ang-(1-7) on dopamine uptake. Interestingly, in the presence of 100 nM losartan (a specific AT1R antagonist), Ang-(1-7) further increased ^3^H-dopamine uptake, suggesting a possible role of AT1R in addition to the Mas receptor participation.

### 3.3. Effects of CNP and Ang-(1-7) on Na^+^, K^+^-ATPase Activity

**Figure 7:**
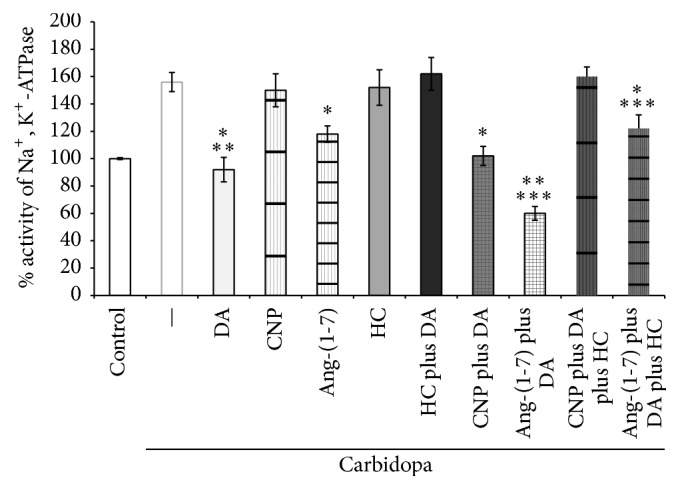
Effects of CNP, Ang-(1-7), dopamine (DA), and hydrocortisone (HC) on Na^+^, K^+^-ATPase activity, calculated as percentage of Na^+^, K^+^-ATPase activity of control values ± SEM in renal cortex. The experiments were carried out in the absence (control) or in the presence of carbidopa. ^*∗*^ *p* < 0.01 compared with carbidopa alone; ^*∗∗*^ *p* < 0.05 compared with Ang-(1-7); ^*∗∗∗*^ *p* < 0.05 compared with dopamine. Number of samples: 8–11.

In order to determine whether the increased uptake of dopamine by CNP or Ang-(1-7) may modify Na^+^, K^+^-ATPase activity in renal tubules, we assayed the effect of CNP and Ang-(1-7) added alone and together with dopamine on the enzyme activity.  Figure 7 shows that, under inhibition of renal dopamine synthesis by carbidopa, Na^+^, K^+^-ATPase activity increased by 56% compared with controls group. Under this condition, when dopamine or Ang-(1-7) was added alone, the enzyme activity diminished approximately by 40% and 25%, respectively, although when dopamine and Ang-(1-7) were added simultaneously, a significant further decrease in Na^+^, K^+^-ATPase was observed (approximately 60% of reduction compared with carbidopa group). On the other hand, the addition of hydrocortisone did not modify* per se* Na^+^, K^+^-ATPase activity as compared with carbidopa treated group, but the corticoid reversed the inhibitory action elicited by dopamine. Moreover, when dopamine and Ang-(1-7) were added simultaneously, hydrocortisone reversed partially the overinhibition elicited by dopamine and Ang-(1-7) on the enzyme activity. Finally, CNP by itself did not show any action on the Na^+^, K^+^-ATPase activity and did not modify the inhibitory action of dopamine on the enzyme activity.

## 4. Discussion

The renal dopaminergic system is a local independent natriuretic system that contributes to preserving a normal balance of sodium and water, blood pressure levels, and renal redox steady state [27]. It regulates sodium homeostasis by inhibiting several sodium transporters, with the Na^+^, K^+^-ATPase being one of the most important enzymes involved in sodium reabsorption in the kidney [17]. However, the clinical efficacy of dopamine infusion has been questioned by a number of clinical studies that indicate that exogenous dopamine lacks effect on promoting renal sodium and water excretion in pathological conditions [28–33]. In this way, we have recently reported that an alteration in dopamine tubular transport by OCTs may decrease dopamine bioavailability into the lumen and impair the interaction of dopamine with D1 and D2 receptors [34]. Therefore, a full activity of OCTs is not only necessary to elicit dopamine diuretic and natriuretic actions when it is exogenously administered to rats but also necessary for ANP to exert its full diuretic and natriuretic effects [34]. Based on this evidence, understanding how Ang 1-7 and CNP can regulate dopamine transport by OCTs and dopamine availability in renal tubules provides new knowledge about the mechanisms that could impair the diuretic and natriuretic effects of dopamine when it is therapeutically administered in patients with renal failure.

In this way, we have previously reported that ANP and URO stimulate dopamine uptake by the tubular cells in the kidney through NPR-A receptors coupled to guanylate cyclase and cGMP as second messenger [20, 21]. As a physiological antagonist, Ang II exhibits the opposite effect through AT1R and PKA and cAMP as second messenger [23]. Our present results show that another important vasoactive peptide, Ang-(1-7), was also able to increase ^3^H-dopamine uptake in renal cortex. The maximum effects observed at 30 minutes of incubation with a concentration of 100 nM Ang-(1-7) are in accordance with the results of previous experiments performed with other vasoactive peptides as ANP, URO, and Ang II [18, 19, 22]. Considering that whole renal tissues were used in the experiments, the neuronal uptake of dopamine was blocked by nomifensine. There are no evidences supporting the presence of dopamine at other extraneuronal sites (such as wall vessels, glomerular, mesangial, and interstitial cells) in a comparable amount with the tubular content of dopamine. Then, in our preparations, the renal tubules are the only structures that are able to uptake significant amounts of dopamine.

Ang-(1-7) effects on dopamine uptake could be mediated by different mechanisms such as the increase of transporters, changes in cell membrane potential, and/or alteration in the carrier affinity. In this sense, renal OCTs have been described to transport dopamine from the bloodstream into the tubular cell [35, 36]. These transporters include OCT-1, OCT-2, and OCT-3 located mainly at the basolateral membrane of proximal tubules cells [37]. OCTs function can be regulated by different protein kinases, since many putative phosphorylation sites can be identified in their intracellular domain [35, 38]. In accordance with the main localization of OCTs in renal cortex, our results showed that the effect of Ang-(1-7) on ^3^H-dopamine uptake was only observed in the renal cortex, but not in medulla. In addition, the basal uptake of dopamine in medulla was even lower than that in renal cortex.

We also evaluated whether CNP, other member of the natriuretic peptide family, might share similar effect to that of ANP or URO on renal dopamine uptake. Our results show that CNP was not able to modify the dopamine uptake even at 45 minutes and also using high concentrations as 100 nM, in both renal cortex and medulla. This lack of effect of CNP on dopamine uptake may be related to different possibilities: CNP exerts its biological effects by selective activation of NPR-B receptors (that are expressed in a low proportion in the kidney), thereby increasing cGMP generation in target cells [39]. In the kidney, CNP stimulates less generation of cGMP than ANP [40, 41]. Thus, the lack of response to CNP stimulation may be due to either an intrinsically low guanylate cyclase activity of NPR-B or the low presence of these receptors in the kidney [42, 43]. In this way, these possibilities are supported by the fact that the administration of high amount of CNP, reaching high circulating levels of the peptide, as those observed in a pathophysiological range, lacked effects on renal hemodynamic and tubular sodium reabsorption and/or excretion [6].

**Figure 8:**
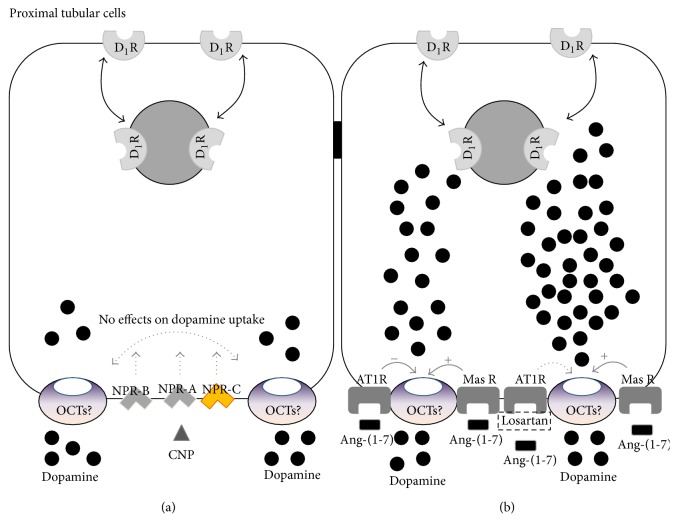
Schematic representation of the mechanism by which CNP and Ang-(1-7) ((a) and (b), resp.) could enhance dopamine tubular transport in proximal tubule cells, by stimulation of extraneuronal uptake. OCTs: organic cationic transporters. Black circles: dopamine; gray triangle: CNP. Black square: Ang-(1-7). Full arrows and +: stimulation; full arrows and −: inhibition; dot arrows: no effect; ?: hypothetical mechanism.

Previous experimental evidences demonstrated that Ang-(1-7) decreased the release and increased the uptake of another catecholamine, norepinephrine, through activation of Mas receptors in the hypothalamus and brainstem of normotensive and SHR [44, 45]. In accordance with this fact, our present results demonstrate that Ang-(1-7) increases dopamine uptake in renal cortex, via activation of Mas receptors. The main receptor for Ang-(1-7) in the kidney is represented by Mas receptor, since the deletion of this receptor abolishes its functional response [46]. Although Ang-(1-7) binds to Mas receptor with a high affinity (Kd 0.83 nM), it is also capable of binding AT1R and AT2R with low-affinity, raising the possibility that certain physiological effects of intrarenal Ang-(1-7) may be mediated by non-Mas receptors [47]. In our experiments, the specific antagonist of Mas receptor, A-779, completely blocked the stimulatory effect of Ang-(1-7) on dopamine uptake, confirming the participation of Mas receptor. Under AT2R inhibition with PD123319, the stimulatory effect of Ang-(1-7) on dopamine uptake remains unchanged, suggesting that AT2R does not affect the action of Ang-(1-7) on the receptor Mas. Interestingly, the stimulatory effect of Ang-(1-7) on dopamine uptake was even more pronounced under AT1R inhibition (70% by Ang-(1-7) plus losartan versus 39% by Ang-(1-7) alone), suggesting that the inhibition of AT1R uncovers an AT1 receptor inhibitory uptake response to Ang-(1-7) and opposing to the Mas receptor stimulatory effect (Figure 8). This result is in accordance with our previous finding, where Ang II inhibited dopamine uptake by stimulation of AT1R [22]. The mechanisms by which Ang-(1-7) through activation of Mas receptor stimulates dopamine uptake could involve several interaction pathways. In this way, a possible interaction of dopamine and Ang-(1-7) with bradykinin (BK), prostaglandins (PGs), and nitric oxide (NO) must be pointed out. A well established interaction between Ang-(1-7) and BK has been shown in blood vessels and neurons, but little is known at renal level [48]. Caruso-Neves et al. demonstrated that BK counteracts the stimulatory effect of Ang-(1-7) on Na^+^, K^+^-ATPase activity [49]. A dopamine-BK interaction has been also suggested. For instance, a dopamine infusion may increase urinary kallikrein activity, sodium excretion, and renal kallikrein mRNA [50, 51]. Urinary kallikrein activity was increased by dopamine infusion in normotensive as well as essential hypertensive patients [52]. Altogether, these data demonstrate that both Ang-(1-7) and dopamine interact with BK. Moreover, PGs and NO have been shown to be involved in Ang-(1-7) and dopamine effects [53–60]. Clark et al. demonstrated that angiotensin-(1-7) exhibits diuretic and natriuretic properties associated with increased production of PGs. The authors suggest that Ang-(1-7) opposes Ang II effects in the kidney, in part through PGs production, which may in turn reduce AT1 receptors [55]. Ang-(1-7) counteracts Ang II hypertensive effects, by binding to Mas receptor and release of NO and PGs. The increase in renal blood flow elicited by Ang-(1-7) in SHR animals was abolished not only by blockade of Mas receptor, but also by inhibition of PGs and NO production, highlighting a close interaction between these systems in the kidney [57–59]. Additionally, Healy et al. have reported that stimulation of D2 receptors by phospholipase A2 (PLA2) increases prostaglandin production in rat inner medullar collecting duct cells [53, 54]. It has been probed that in renal medulla and cortex dopamine increases nitric oxide synthase (NOS) activity [61]. On the other hand, administration of L-NAME reduced the natriuresis and diuresis elicited by dopamine and decreased urinary nitrate excretion, indicating NO involvement in regulation of renal sodium excretion, and, besides, NO system is required for the full expression of diuretic and natriuretic responses elicited by D1 receptor activation [62]. We have also reported that while inhibition of particulate cGMP inhibits ANP and URO stimulating effects on dopamine uptake in renal tissue, inhibition of soluble cGMP by ODQ lacked effects, suggesting that ANP and URO effects are nondependent on NO [20, 21]. Brouwers et al. have reported that AT2 receptors may have a pathophysiological role in modulating renal hemodynamic effects of Ang II in SHR rats [60]. By using captopril (ACE inhibitor), compound 21 (AT2 receptor agonist), fenoldopam (D1 receptor agonist), PD123319 (AT2 receptor antagonist), L-NMMA (NOS inhibitor), indomethacin (COX inhibitor), and icatibant (B2 receptor antagonist), they showed a complex and intricate interaction and relationship between Ang II, Ang-(1-7), dopamine, BK, NO, PGs, and cyclooxygenase systems in the kidney that, beyond regulation of physiological hemodynamic of kidney, participates in the development of hypertension. Taking into account all these evidences, we cannot discard BK, PGs, or NO involvement in Ang-(1-7) effects on renal dopamine uptake and further experiments should be performed to test this hypothesis.

The natriuretic peptides exert their biological actions through activation of NPR-A and/or NPR-B receptors [4]; meanwhile, the clearance of these peptides depends on their binding to NPR-C receptors [63]. In our experiments, the specific inhibitor of NPR-A, anantin, did not affect* per se* dopamine uptake in renal cortex and CNP lacked effects on the same process even in the presence of anantin. In order to discard the involvement of clearance receptors NPR-C, as the cause of the lack of action of CNP on dopamine uptake, we performed another set of experiments in the presence of the truncated peptide cANP (4-23-amide). This peptide binds to NPR-C receptors, which are devoid of kinase and guanylyl cyclase activities [64]. The analogous cANP (4-23-amide) did not alter dopamine uptake even alone or in the presence of CNP. Altogether, these results suggest that CNP has no effect on renal dopamine uptake through activation of NPR receptors (Figure 8).

When comparing the potency of Ang-(1-7) with other vasoactive peptides that stimulate renal dopamine uptake (Figure 4), Ang-(1-7) displayed more potency than ANP (39% versus 32%, resp.) but lower than URO (39% versus 45%). However, when we evaluated the effects of Ang-(1-7) under AT1R inhibition with losartan, the stimulatory action of Ang-(1-7) on dopamine uptake rose up to 70%.

In order to elucidate whether CNP or Ang-(1-7) effects on dopamine uptake may modify renal sodium excretion, we determined the effects of these vasoactive peptides on the Na^+^, K^+^-ATPase activity. To avoid possible effects of endogenous renal dopamine and neuronal dopamine, we employed carbidopa to inhibit renal dopamine synthesis and nomifensine to prevent neuronal dopamine uptake. Renal Na^+^, K^+^-ATPase activity increased when dopamine synthesis was inhibited by carbidopa, in agreement with the decrease of dopamine availability. Under this condition, when exogenous dopamine was added, the activity of the enzyme decreased, according to the restored dopamine availability. On the other hand, Ang-(1-7) alone decreased significantly the sodium pump activity, and when it was added together with dopamine, they decreased further Na^+^, K^+^-ATPase activity, showing an additive effect on the sodium pump. To assess whether Ang-(1-7)-induced inhibition of Na^+^, K^+^-ATPase activity is related to Ang-(1-7) stimulation of extraneuronal uptake of dopamine, additional experiments were performed in the presence of hydrocortisone, a known inhibitor of extraneuronal uptake of amines. Hydrocortisone by itself reproduced similar levels of Na^+^, K^+^-ATPase activity as carbidopa but reversed the overinhibition of the enzyme induced by Ang-(1-7) plus dopamine. This finding confirms that Ang-(1-7) may stimulate dopamine uptake in the kidney, by regulating a typical hydrocortisone sensitive-extraneuronal uptake of amines.

It must be pointed out that renal effects of Ang-(1-7) are complex to understand and may not always be opposite to those elicited by Ang II. In fact, Ang-(1-7) effects may be different and dependent on (a) the animal models used in the research; (b) the concentration reached by Ang-(1-7) at local or systemic levels; (c) the nephron segment where the study was performed; (d) the level of RAS activation; and (e) the sodium and water status in the kidney [65]. Several studies carried out* in vitro* and also* in vivo* have suggested that Ang-(1-7) increases natriuresis and diuresis by regulating the activity of sodium transporters in the proximal tubule [13]. It has been described that Ang-(1-7) inhibits the ouabain-sensitive Na^+^-K^+^-ATPase in isolated rat proximal tubules [66]. Moreover, other authors have reported that Ang-(1-7), at high concentrations (10^−8^ M), inhibits fluid absorption in isolated rat proximal straight tubules [67]. These reports agree with our results and show how Ang-(1-7) can inhibit Na^+^-K^+^-ATPase activity in the kidney. On the contrary, López Ordieres et al. showed that rat kidney Na^+^-K^+^-ATPase activity decreased roughly by 40–70% with 10^−10^ to 10^−6^ M Ang-(1-7) but increased by 22% with 10^−12 ^M peptide concentration, thus indicating a biphasic effect [68]. In this sense, Pinheiro et al. have demonstrated that Ang-(1-7) may exert antidiuretic and antinatriuretic effects, especially in water-loaded animals [69, 70]. With regard to glomerular level, some reports showed that Ang-(1-7), when it was infused in rats at low doses, could vasodilate preconstricted renal afferent arterioles in rabbits and increase renal blood flow [58, 71]. This action differs from that of Ang II in the same vessels, since Ang II has shown higher reactivity in efferent arterioles, without changes in afferent arteriolar resistance [72].

Unlike Ang-(1-7), CNP, by itself, did not affect the Na^+^-K^+^-ATPase activity. Moreover, when CNP was added together with dopamine, the natriuretic peptide showed an inhibitory effect on renal Na^+^, K^+^-ATPase as that observed when dopamine was used alone. These results demonstrate that CNP lacks regulating actions on Na^+^, K^+^-ATPase activity.

The natriuretic and diuretic effects of dopamine in the kidney are mediated by stimulation of D1 receptors, which are mainly intracellular and located in basal conditions [17]. These receptors can be recruited to the plasma membrane, either by D1 agonists or by the increase of intracellular dopamine availability [17]. It was also shown that the second messenger, cGMP, causes a rapid translocation of D1 receptors to the plasma membrane [17]. On the other hand, Mas receptor activation leads to nitric oxide generation, which can increase intracellular cGMP levels through activation of the soluble form of guanylate cyclase [12, 73]. Therefore, when Ang-(1-7) increases the renal uptake of dopamine, it also induces generation of cGMP, two factors closely involved in the recruitment of D1 receptors to the plasma membrane, thus helping to sustain the response to dopamine and providing a general mechanism by which peptide hormones can regulate indirectly sodium homeostasis, via sensitization of dopamine receptors.

A complex interaction between AT1 and AT2 and Mas receptors should be taken into account to understand the sense of our results. Whether the increase in ACE2 and Ang-(1-7) might contribute to the beneficial effects of classical RAS inhibitors is still a matter of debate. In this sense, Ferrario et al. reported that treatment with losartan enhanced plasma and urinary concentrations of Ang-(1-7) and ACE2 activity in renal cortex, with no changes in the expression of ACE and in Mas and AT1 receptors [74]. Therefore, the increase of ACE2 activity by AT1 blocking will enhance renal Ang-(1-7) concentration, which, in turn, can increase dopamine uptake and activation of Mas receptor, as we have observed in our experiments. Thus, the increasing in dopamine transport in renal cortex, elicited by Ang-(1-7), together with the increasing activity of ACE2-Ang-(1-7)-Mas receptor axis, contributes to renoprotection triggered by AT1 receptor blockers. This is in agreement with Da Silveira et al. who reported that, in a murine model [75], exogenous activation of Mas receptor by the agonist AVE 0991 protects against adriamycin-induced nephropathy and contributes to the beneficial effects of AT1 receptor blockade with losartan. In addition, the administration of the Mas receptor antagonist, A-779 in experimental models of hypertension, attenuated the effects of ACE inhibitors and Ang II receptor blockers [76]. Moreover, Igase et al. demonstrated, in a model of hypertensive nephropathy, that the inhibition of AT1 receptors by olmesartan increased plasma levels of Ang-(1-7), leading also to cardio- and renoprotective effects by reducing glomerular sclerosis and renal perivascular collagen deposition [77]. In the same way, Zong et al. showed that rats with adriamycin-induced heart failure had low plasma levels of Ang-(1-7) and reduced expression of myocardial Mas receptor, while treatment with telmisartan and losartan restored Ang-(1-7) levels and suppressed myocardial AT1 receptor expression but did not influence the expression of Mas and AT2 receptors [78]. Sukumaran et al. have reported that treatment with losartan in Lewis rats with autoimmune myocarditis increased also myocardial levels of ACE2 and of Mas receptor and reduced fibrosis and hypertrophy and marker molecules of inflammation, collagens I and III, and atrial natriuretic peptide [79]. These results indicate that AT1 blockage significantly improved left ventricular function and ameliorated the progression of cardiac remodeling through the modulation of ACE-2/Ang-(1-7)/Mas receptor axis. On the other hand, Kocks et al. reported that healthy individuals receiving ACE inhibitors combined with low sodium diet exhibit elevated plasma levels of Ang-(1-7) without changes in Ang II concentration [80]. Consequently, the combination of ACE inhibition and a low sodium diet appeared to shift the balance between Ang-(1-7) and Ang II towards Ang-(1-7), which, in turn, might contribute to the therapeutic benefits of ACE inhibition. Da Silveira et al. have reported that treatment with the Mas receptor agonist AVE 0991 improved renal function, reduced urinary protein loss, and attenuated histological changes [75]. Similar renoprotection was observed after treatment with the AT1 receptor antagonist, losartan, which reestablished Mas receptor expression and ACE2 expression. But the same authors demonstrated that the presence of Mas receptor seemed to be critical for the renoprotective effects of AT1 receptor antagonists, since the treatment with losartan was not able to attenuate adriamycin-induced renal injury in Mas knockout mice.

In conclusion, the knowledge of the role that ACE2/Ang-(1-7)/Mas axis plays in the context of renal disease may be useful to prevent acute and chronic inflammation and fibrosis medications which target specifically the ACE2/Ang-(1-7)/Mas axis may offer new therapeutic opportunities to treat human nephropathies [81, 82]. Taken together, these findings indicate that the increased activity of the ACE2-Ang-(1-7)-Mas receptor axis contributes to the cardio- and renoprotection triggered by ACE inhibitors and ARAs. These complex interactions between AT1 and Mas receptors should be taken into account for the interpretation of the results.

In summary, Ang-(1-7) stimulates dopamine uptake in renal cortex through activation of Mas receptor. Unlike other members of natriuretic peptides, CNP was not able to regulate renal dopamine uptake and Na^+^, K^+^-ATPase activity. Dopamine and Ang-(1-7) may act in concert to inhibit tubular sodium reabsorption, as a consequence of increased dopamine uptake, resulting in overinhibition of Na^+^, K^+^-ATPase activity. In this way, Ang-(1-7) opposes one of the mechanisms of sodium transport regulation exerted by Ang II. Further studies must be performed in order to determine the intracellular events and signaling involved in Ang-(1-7)-dopamine relationship in the kidney. Our results demonstrate the existence of a close relationship between renal dopamine and vasoactive peptides, an interaction that might impact hydrosaline balance and whose impairment could be also involved in the pathogenesis of arterial hypertension.
